# Assessing the quality of care in primary health care facilities in all municipalities in Kosovo 2016–2023

**DOI:** 10.3389/fpubh.2025.1596249

**Published:** 2025-07-02

**Authors:** Astrid M. Knoblauch, Sarah Rajkumar, Robert Canavan, Qamile Ramadani, Ariana Bytyçi Katanolli, Shegë Bahtiri, Debra Stevenson, Merita Stavileci Mustafa, Nicu Fota, Jana Gerold

**Affiliations:** ^1^Swiss Tropical and Public Health Institute, Swiss Center for International Health, Allschwil, Switzerland; ^2^University of Basel, Basel, Switzerland; ^3^Acessible Quality Healthcare Project, Pristina, Kosovo; ^4^Embassy of Switzerland, Swiss Cooperation Office, Pristina, Kosovo

**Keywords:** Kosovo, quality of care, infrastructure, clinical observations, patient satisfaction, primary healthcare, non-communicable diseases

## Abstract

**Introduction:**

Access to quality primary healthcare (PHC) in Kosovo is progressive with substantial reforms that have been implemented to promote the health of its people since its independence in 2008. However, substantial challenges remain, particularly among managing non-communicable diseases (NCD) like diabetes and hypertension, due to gaps in structural and procedural aspects. The Accessible Quality Healthcare project (2016–2027) aimed at improving the health of the Kosovar population by strengthening the quality of PHC services with a specific focus on NCDs. Three quality of care studies were conducted with the aim of measuring progress in PHC and NCD management.

**Methods:**

Three facility-based, cross-sectional studies were conducted in 2016, 2018 and 2023 across 12, 20, and 38 selected municipalities in Kosovo, respectively. Data were collected on PHC infrastructure, provider-patient interactions, and patient outcomes using standardized assessments, observational tools, and exit interviews.

**Results:**

The overall infrastructure score improved from 56% in 2016 to 68% in 2023. Clinical observation scores comprised five categories. For instance, doctors’ knowledge and compliance with clinical history and physical examinations revealed a marked increase overall from 2016 to 2023, hygiene and infection prevention control measures increased steadily in 14 municipalities but remained below 60% in 16 municipalities. Clinical adherence to guidelines for managing diabetes and hypertension improved markedly but remained low overall (from 36% in 2016 to 60% in 2023 with regards to diabetes and 35% in 2016 to 58% in 2023 regarding hypertension). The overall experience of patients exiting facilities was positive and their perception of doctors’ behaviour scored more than 60% in all years and in seven of the eight indicators. Negative exceptions included a drop in explanations regarding medicine intake from 96% in 2016 to 56% in 2023 and regarding the doctors’ verbal anamnesis, physical examination and explaining the health problems (from 97% in 2016 to 77% in 2023).

**Conclusion:**

While marked progress was made in improving Kosovo’s healthcare infrastructure and clinical practices, persistent disparities in municipal performance and gaps in doctor-patient communication highlight the need for targeted interventions. Continued investment in healthcare worker training, better adherence to clinical protocols, and improved communication will be essential to sustain progress and align Kosovo’s healthcare system with European Union standards.

## Introduction

Kosovo, as a middle-income country, has been a progressive economy but remains one of the poorest countries in Europe (1–3). Unemployment remains a challenge with an unemployment rate of 33% in 2022—one of the highest rates in Europe and the Balkans. Kosovo has one of the youngest populations in Europe with an average age of 26, however, the youth unemployment rate stood at almost 60% in the same year (3).

In terms of population health, life expectancy at birth has had a positively increasing trajectory from 74 years in 2000 to 77 in 2021 and 80 in 2022, bringing Kosovo in line with those in western Europe (>80) and higher than Albania (77), Bosnia Herzegovina (75) and Georgia (72), for instance (4). Albeit disease prevention is improving in Kosovo, unhealthy lifestyle factors, such as poor nutrition, alcohol consumption, physical inactivity, obesity and smoking are still prevalent (5–9). This is despite stringent measures imposed to control some of those factors, such as prohibiting indoor smoking, banning tobacco advertising and promoting physical activity (10). They contribute to the heavy non-communicable disease (NCD) burden that continues to be reported in Kosovo and reinforces the concerning findings from the World Health Organization (WHO)‘s “Primary health care in Kosovo: rapid assessment” in 2019 (11), which reported that in 2015, hospitalization rates for diabetes stood at 396 per 100,000 inhabitants and 776 per 100,000 for hypertension. The average hospitalization rates for the 33 Organization for Economic Cooperation and Development countries were, by comparison, less than half for diabetes (174 per 100,000) and a third for hypertension (131 per 100,000) (11).

Kosovo’s healthcare system was based on the former Soviet Union (1922–1991) ‘Semashko model’, a state funded purchaser and provider of healthcare services for all citizens (12). Since the post-Soviet transition, Kosovo has struggled with an under-performing health system, which has not adequately met the health-related needs of its citizens. Care processes continued to be geared towards treating communicable diseases and failed to adapt to evolving population needs with the growing burden of non-communicable diseases (NCDs). Therefore, it has been permanently undergoing major healthcare system reforms with the aim of providing decentralized services and extending health insurance coverage to all residents. Kosovo’s European Union (EU) membership application in 2022 indicates that their stance on any reforms would advance in alignment with EU healthcare policies to (a) protect and improve the health of EU citizens; and (b) support the modernisation and digitalisation of health systems and infrastructure (13, 14). Furthermore, Kosovo has taken positive steps to embrace the United Nations Sustainable Development Goals (SDGs), despite not being a signatory due to its status as a non-member (1).

The primary health care (PHC) setting can be instrumental in improving NCD prevention by tackling patients’ unhealthy social habits with, among others, motivational counselling on life style behaviour before the onset of clinical disease (8, 11, 15–17). WHO emphasises using a PHC approach owing to its inclusive, equitable, cost-effective and efficient nature (18). Moreover, the quality of care in PHC services is crucial for achieving the SDG3 targets, universal health coverage and encouraging the community to benefit from the services provided. However, Kosovo faces several challenges at all levels setting itself on course for attaining these goals and realizing its efforts (19). Previous studies in Kosovo revealed that the Kosovar citizens were less satisfied with public PHC visits than in other European countries (11, 20). The low physician to population rate, (1.5 per 1,000 compared to the EU average of 4.3 per 1,000) (21), the lacking competencies of a demoralised staff (22), the expectation of medical equipment that is lacking or the out-of-pocket payments (14) are all potential components for dissatisfaction that provide plausible reasons for shunning PHC services in favour of secondary/tertiary care or private services (11).

In 2015, Kosovo welcomed the launch of the “Accessible Quality Healthcare” (AQH) project, a project of the Swiss Agency for Development and Cooperation (SDC) and facilitated by the locally registered office of the Swiss Tropical and Public Health Institute (Swiss TPH) with national partners. The project’s design was intended to improve the health of the Kosovar population by strengthening the quality of PHC in the public sector, with a specific focus on NCDs (7, 8, 23). The long-term AQH project (divided into three phases from 2016 to 2027) supports national institutions in reforming their health sector by assisting the decentralization of PHC services through a health system strengthening approach. The three interlinked areas of support are the following: (1) PHC providers deliver quality services that respond better to communities’ needs, (2) health managers improve their performance in guiding service delivery towards continuous quality improvement, and (3) the population improves its health literacy and is empowered to demand the right to quality services and better access to care. The AQH support covered in the first phase approx. 25% of the population (12 municipalities), in the second phase more than 50% of the population (20 municipalities) and, in the third phase, aims for a national coverage (38 municipalities).

Swiss TPH conducted, as part of the above outlined AQH intervention, a quality of care study in 2016 (12 municipalities), in 2018 (all 38 municipalities) and in 2023 (20 municipalities). The aim of these studies was to measure project progress and document the changes induced in PHC by comparing the quality of care of the structural (facility infrastructure) and procedural (provider-patient interaction) aspects, together with selected outcomes (patient experience after consultation) in several PHC centres in each of the selected municipalities.

## Methods

### Study framework and survey design

Three facility-based, cross-sectional surveys were conducted focusing on the quality of care in the PHC services across selected Kosovo municipalities. They focused on patients with cardiovascular diseases, including hypertension, diabetes and asthma. These surveys were based on the framework provided by Donabedian (24, 25), which was also used in similar studies (26, 27).

Data were collected at three different levels: (i) the healthcare facility infrastructure (structural attributes), (ii) the health care provider (process attributes), and (iii) the patient (outcome attributes). At health facility level, the quality of infrastructure was assessed by means of a health facility assessment tool. At health provider level, the quality of provider-patient interactions was assessed through observations with an observational tool, and at patient level, the quality of patient experience after consultation was assessed via exit interviews.

### Sampling and inclusion

The decentralized PHC system in Kosovo provides one main family medicine centre (MFMC) and several family medicine centres (FMC) in each municipality. MFMCs are the largest of the PHC facilities offering the most services, staff and medical equipment and have a higher volume of patients compared with FMCs.

Inclusion criteria for health facilities required that (i) the facilities were either a MFMC or FMC; (ii) they had a minimum of one medical doctor assigned to the facility for at least 1 day per week; and (iii) they were supported by AQH project activities. Inclusion criteria for health providers from the selected facilities for provider–patient observations required that (i) the health providers were general practitioners providing PHC services; (ii) the patients were at least 18 years old or accompanied by a legal guardian, accessing the facility and receiving a consultation from a health provider; (iii) oral informed consent was given by the provider; and (iv) oral informed consent was given by the patient or his or her legal guardian. Inclusion criteria for patients accessing the selected facilities and participating in exit interviews were that (i) they were patients of at least 18 years or accompanied by a legal guardian, accessing the facility and received a consultation from a health provider; (ii) oral or written informed consent was given by the patient or his or her legal guardian; and (iii) they were accessing the facility to receive services either for themselves or their accompanying minors.

A random probability proportional to size sampling procedure was applied for each of the health facilities with the assumption that the facilities would be allocated to urban and rural areas proportional to the size of urban and rural populations in the region. The assumption is based on the central institutional planning of rural and urban health facilities per population. Medical doctors observed in the provider–patient consultations were selected randomly among all medical doctors practicing in a facility. All doctor-patient consultations that occured during data collection in a health facility (i.e., 1 day) were observed, depending on the capacity of the data collectors. Thus, the same doctor could be observed repeatedly. For the exit interviews, all patients at the facility who received care either for themselves or for a child in their care were interviewed upon exiting the facility, provided they gave consent and depending on the capacity of the data collectors. The patients included for exit interviews were not necessarily the same as for the provider–patient observation.

### Data collection

The quality of care assessment framework was based on three tools. The first: The infrastructure questionnaire contained six dimensions such as facility infrastructure, overall cleanliness and maintenance and hygiene and safety standards. The second: Observations on provider—patient interactions contained three dimensions such as general aspects on adherence on principles of clinical history and physical examination and the third: Patient experience on exit interviews contained four dimensions including satisfaction with privacy, satisfaction with doctor-patient interactions, satisfaction with the quality of the facility and the socio-demographic and economic aspects.

The survey considered a mix of indicators from the WHO “Service Availability and Readiness Assessment”-tool and the “Tool to Improve Quality of Health Care” within the “ACCESS” program supported by the Novartis Foundation for Sustainable Development (2014) (28, 29). The tools were adapted in collaboration with national partners to the Kosovo local context thereby taking into consideration the national PHC norms or, where these were unavailable, the WHO norms established in the “Package of Essential NCD Interventions” (30).

Data collection was performed electronically using the Open Data Kit (ODK) software on portable electronic tablet devices. During and after each day of data collection, the local study coordinator and the supervisors conducted quality assurance. This entailed monitoring how many observations were conducted at each facility and checking samples of the questionnaires for completeness and consistency. The local study coordinator ensured that the data was correctly saved and transferred on a daily basis to a secured server at Swiss TPH in Basel, Switzerland.

In 2016, the data collection team consisted of 12 members divided into 6 gender balanced sub-teams wherever possible. In 2018, the data collection team consisted of 23 data collectors divided into 10 sub-teams. In 2023, the data collection team consisted of 12 members divided into 6 sub-teams. The sub-teams were gender balanced, which facilitated implementation in the field. Most data collectors had a medical or public health background in addition to previous survey experience. Across all surveys, medical students acted as observers for the clinical consultations and were trained and received clear instructions prior to data collection. Each sub-team was assigned to a set of designated facilities where they conducted the assessments closely monitored by the local study coordinator and supervisors.

Data collection started at the opening hour of the health facility (typically at 8 am) and provider–patient observations and exit interviews were conducted until the closing of consultations at the facility (usually between 14:30–15:00 h) or until the end of the shift of the doctor. Two interviewers were present at each site conducting clinical observations and the exit interviews, respectively. Exit interviews were conducted in an appropriate location ensuring maximum privacy and confidentiality. The structural aspects of the facility were assessed when the facility was closed or at the end of a working shift.

### Data analysis

For each domain (i.e., infrastructure, clinical consultation and exit interview), an overall score was calculated where the denominator was ‘total number of questions,’ and the nominator was ‘number of correctly answered questions.’ The latter could refer to (1) the availability and/or functionality of infrastructure, equipment or drugs in the infrastructure assessment; or (2) doctors’ adherence to standards and protocols according to good practice for the doctor-patient observations. These were sub-divided into five categories: (i) principles of clinical history and physical examinations, (ii) hygiene, infection prevention and control, (iii) clinical assessment of a diabetes mellitus patient, (iv) clinical assessment of a patient with arterial hypertension and (v) clinical assessment of a patient with other condition than diabetes mellitus or hypertension; and (3) satisfying/positive answers with regards to quality of care from the exiting patients in the exit interviews. In addition, summary scores of sub-categories within the different domains were calculated using the same approach. Scores were typically stratified per municipality and year.

The overall scores were calculated as additive indices to indicate the achieved percentage score. For a certain set of questions, e.g., infection prevention and control measures, the additive index counts the answers/criteria which were fulfilled or not fulfilled. Questions/criteria which were not applicable were not considered. The number of positive answers was then divided by the total number of valid answers (ratio). This way a percentage score was obtained for each patient.

Data were analysed using Stata Statistical Software v12.1 (2016), Stata Statistical Software/SE v15.0 (2018) and Stata Statistical Software and R statistical software in 2023. To calculate the overall scores for each dimension, weights reflecting the relative importance of each indicator and work stream were assigned. These weights were determined based on similar studies and in agreement among the AQH team. Summary cross-tables outline differences between MFMC and FMC, municipalities, rural and urban facilities, as well as gender of the patient whereby the different PHCs were categorized into one MFMC and two FMCs according to their function.

Statistical differences were assessed based on 95% confidence intervals (CIs), corresponding to a 5% significance level.

### Ethical considerations and clearance

Participation in the study was voluntary. Participants were given an information leaflet and asked for their written consent before the interviews and observations took place. Participants were informed that (i) their participation was voluntary; (ii) they could withdraw from participation at any time without consequences; and (iii) non-participation would not have any negative effects. Participants were also informed how the data would be used and that confidentiality was ensured as no names or other identifying aspects would be collected.

Ethical clearance was obtained in the 2016 study from the Board for Ethical-Professional Supervision on 13th April 2016 (Reference Number: 02/2016). For this purpose, the Ministry of Health (MoH) was informed about the objectives of the study and the study design as well as the content of the data collection tools. In the 2018 study, ethical clearance was obtained from the Kosovo Medical Chamber on 3rd of August, 2018 (Reference Number: 04/2018). In the 2023 study, ethical clearance was obtained from the Kosovo Medical Chamber on 24th April 2023 (Reference Number: 66/2023).

## Results

### Infrastructure

**Figure 1:**
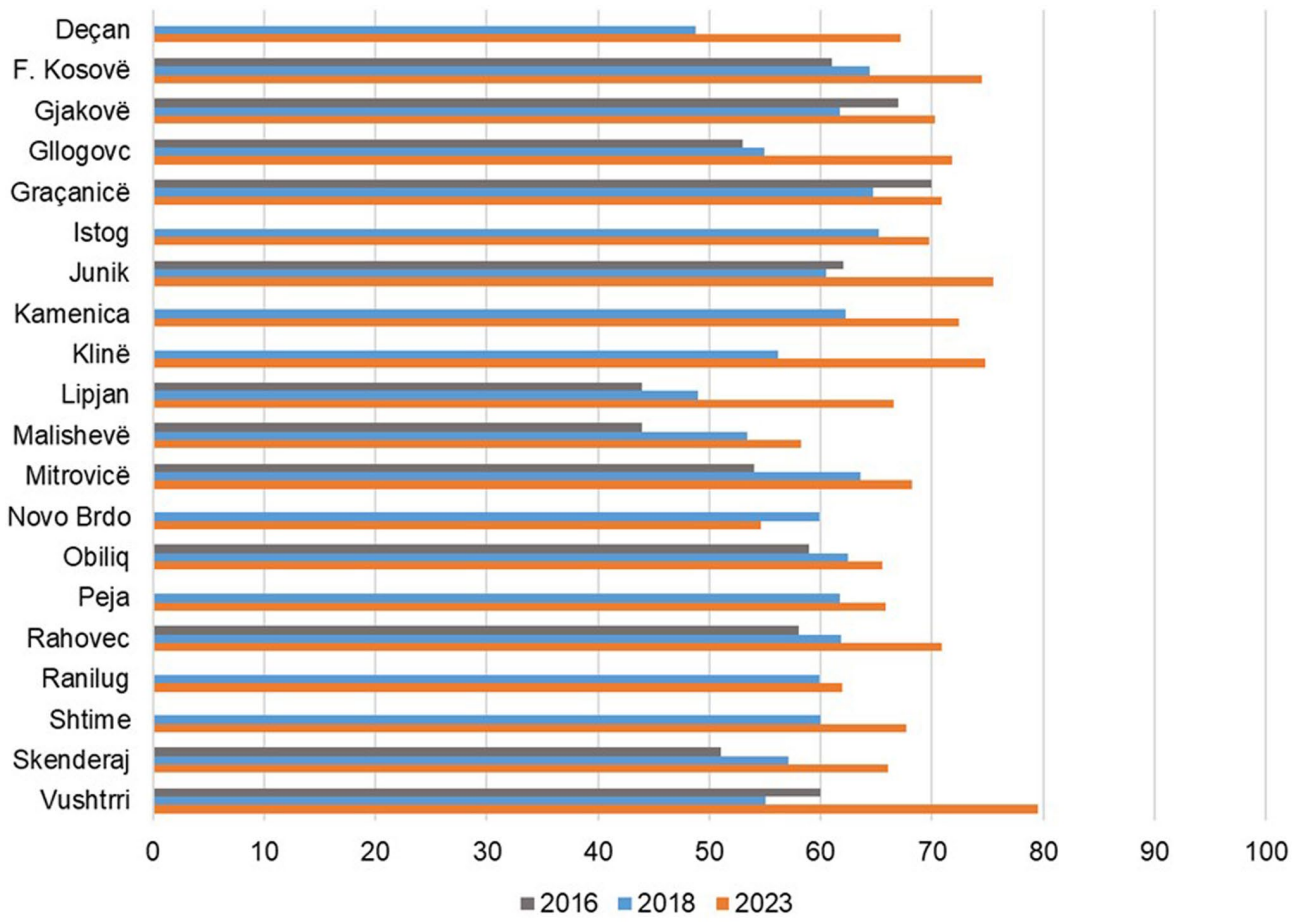
Overall infrastructure score by municipality 2016, 2018, and 2023.

The trend for the combined overall average infrastructure scores from 2016 (56%) to 2018 (59%) and finally to 2023 (68%) was positive across the study facilities in almost all municipalities with the exception of the Novo Brdo municipality, which showed a slight downward trajectory from 60% in 2018 to 55% in 2023. These overall scores included both MFMCs and FMCs (Figure 1).

The overall average infrastructure score in MFMCs remained stable between 2016 (71%) and 2018 (70%) and then increased to 80% in 2023. In FMCs, there was a steady increase from 51% in 2016 to 60% in 2018 and to 69% in 2023 (Table 1).

**Table 1:** Infrastructure scores stratified by facilities in 2016, 2018, and 2023.

	MFMC (mean, 95% CI)			FMC (mean, 95% CI)		
	2016 (*n* = 11)	2018 (*n* = 19)	2023 (*n* = 18)	2016 (*n* = 11)	2018 (*n* = 35)	2023 (*n* = 34)
Overall infrastructure	71 (65–78)	70 (67–74)	80 (76–83)	51 (47–55)	60 (57–63)	69 (66–71)
Facility Infrastructure & Cleanliness	88 (84–92)	85 (82–89)	90 (87–94)	64 (58–69)	62 (57–67)	74 (70–78)
Hygiene	83 (75–92)	79 (71–87)	92 (87–97)	73 (67–80)	68 (63–74)	93 (91–95)
Public Accountability	83 (73–92)	87 (80–94)	89 (82–95)	64 (56–71)	71 (63–78)	66 (58–73)
Guidelines & Material	89 (81–97)	68 (58–78)	89 (79–99)	49 (38–60)	56 (47–65)	78 (70–85)
General Medical Equipment	69 (58–80)	65 (56–74)	82 (77–87)	45 (37–53)	60 (55–65)	72 (69–75)
Availability of Medicines	69 (58–80)	61 (56–66)	65 (59–70)	55 (52–57)	53 (51–56)	54 (50–57)

Improvements in 2023 were found in most of the following infrastructure sub-categories where the average percentage infrastructure score was assessed: (i) facility infrastructure and overall cleanliness; (ii) hygiene; (iii) public accountability; (iv) guidelines and materials; v) general medical equipment; and (vi) availability of medicines. Facility infrastructure and cleanliness trends were mostly positive and values were generally quite high (overall average >80% in MFMCs and >60% in FMCs). Hygiene standards dramatically increased overall. They were, however, higher in 2016 than in 2018 but improved again in 2023. The public accountability sub-category had varying results depending on the items assessed. In regard to visibility of the contact phone number displayed to the public, there was an increase from an overall average of 33% in 2016 to 50% in 2023. In addition, there was an increased availability of information leaflets in facilities concerning the MoH helpline for citizens’ complaints from an overall average of 46% in 2016 to 87% in 2023. Furthermore, the trend for displaying posters from pharmaceutical companies saw a dramatic decrease from an overall average of 80% in 2016 to 17% in 2023. The availability of guidelines and material improved in 10 of 12 municipalities that had data for both 2016 and 2023, 2 of those 10 (Mitrovicë and Malishevë) more than doubled their 2016 score. The availability of general medical equipment improved in 11 of 12 municipalities that had data for both 2016 and 2023, however, the availability of medicines improved in only 6 of 12 municipalities that had data for both 2016 and 2023. In addition, stratifying facility types, the availability of medicines in MFMCs was lower in 2023 (with an overall average of 65%) than in 2016 (69%). FMCs had a steady overall average of 54% in 2023 compared to 55% in 2016.

### Clinical Observations

In 2016, 1,013 clinical observations were conducted, 566 in MFMCs and 447 in FMCs. In 2018, 1,116 clinical observations were made, 644 in MFMCs and 472 in FMCs and in 2023, 922 clinical observations were made, 585 in MFMCs and 337 in FMCs.

The average number of observations per facility in 2016 was 26 (min: 0; max: 112), 23 in 2018 (min: 2; max: 98) and 18 in 2023 (min: 2; max: 108).

In 2016, 14% of patients attended the facility for hypertension, 3% for diabetes and 67% for reasons other than hypertension and diabetes, 16% came for a referral. In 2018, 10% of patients attended the facility for hypertension, 3% for diabetes and 67% for reasons other than hypertension and diabetes, 20% came for a referral. In 2023, 14% of patients attended the facility for hypertension, 6% for diabetes and 65% for reasons other than hypertension and diabetes, 15% came for a referral.

The clinical observation scores from the municipalities that were surveyed in 2016, 2018 and 2023 were compared and the results presented as overall average percentages. They comprised the following five categories: (i) principles of clinical history and physical examinations; and (ii) hygiene and infection prevention and control; (iii) clinical assessment of a diabetes mellitus patient; (iv) clinical assessment of a patient with arterial hypertension; and (v) clinical assessment of a patient with other condition than diabetes mellitus or hypertension.

**Figure 2:**
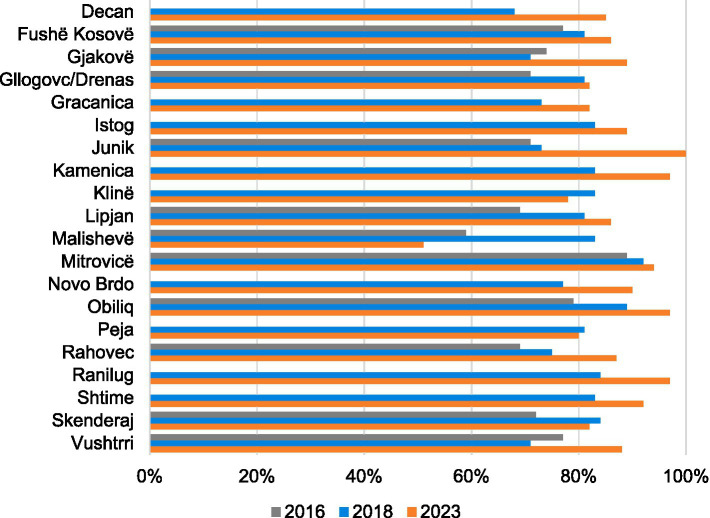
Clinical observation scores for doctors’ knowledge and compliance with principles of clinical history and physical examinations, by municipality (2016, 2018, and 2023).

The doctors’ knowledge and compliance results revealed marked increases in almost all municipalities (Figure 2). The only substantial decrease in the overall average, from 83% to 52%, occurred in Malishevë. Klinë, Peja and Skenderaj were the only other municipalities that saw a slight negative trajectory from 2018 to 2023. The criteria under observation for doctor’s hygiene, infection prevention and control measures included measures such as whether the doctors washed their hands before and after procedures, applied proper decontamination procedures and wore protective gloves and masks when required. In 2016, very few measures for the prevention and control of infection were applied during the observed consultations; an average of only 8% of infection control measures were employed over all facilities and there was no difference between rural and urban facilities.

**Figure 3:**
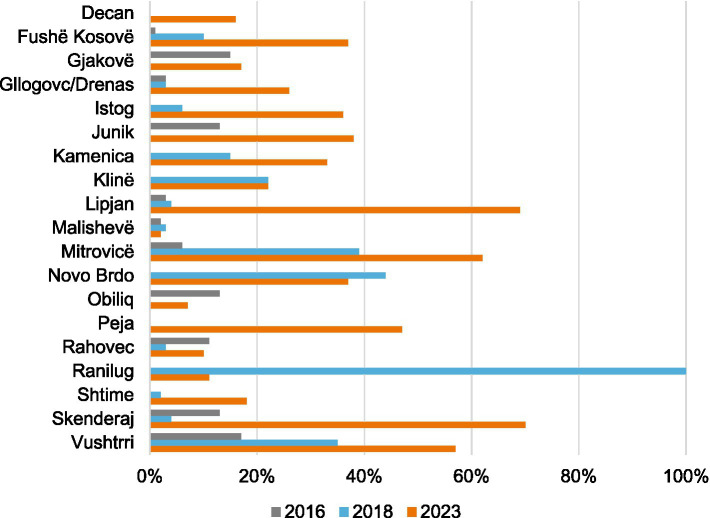
Clinical observation scores by municipality (2016, 2018, and 2023). Doctors’ hygiene and infection prevention control measures.

Infection prevention and control values for 2016, 2018 and 2023 increased steadily in 14 municipalities but nevertheless remained low in many of them and decreased by almost 90% in Ranilug from 2018 to 2023 (Figure 3).

**Figure 4:**
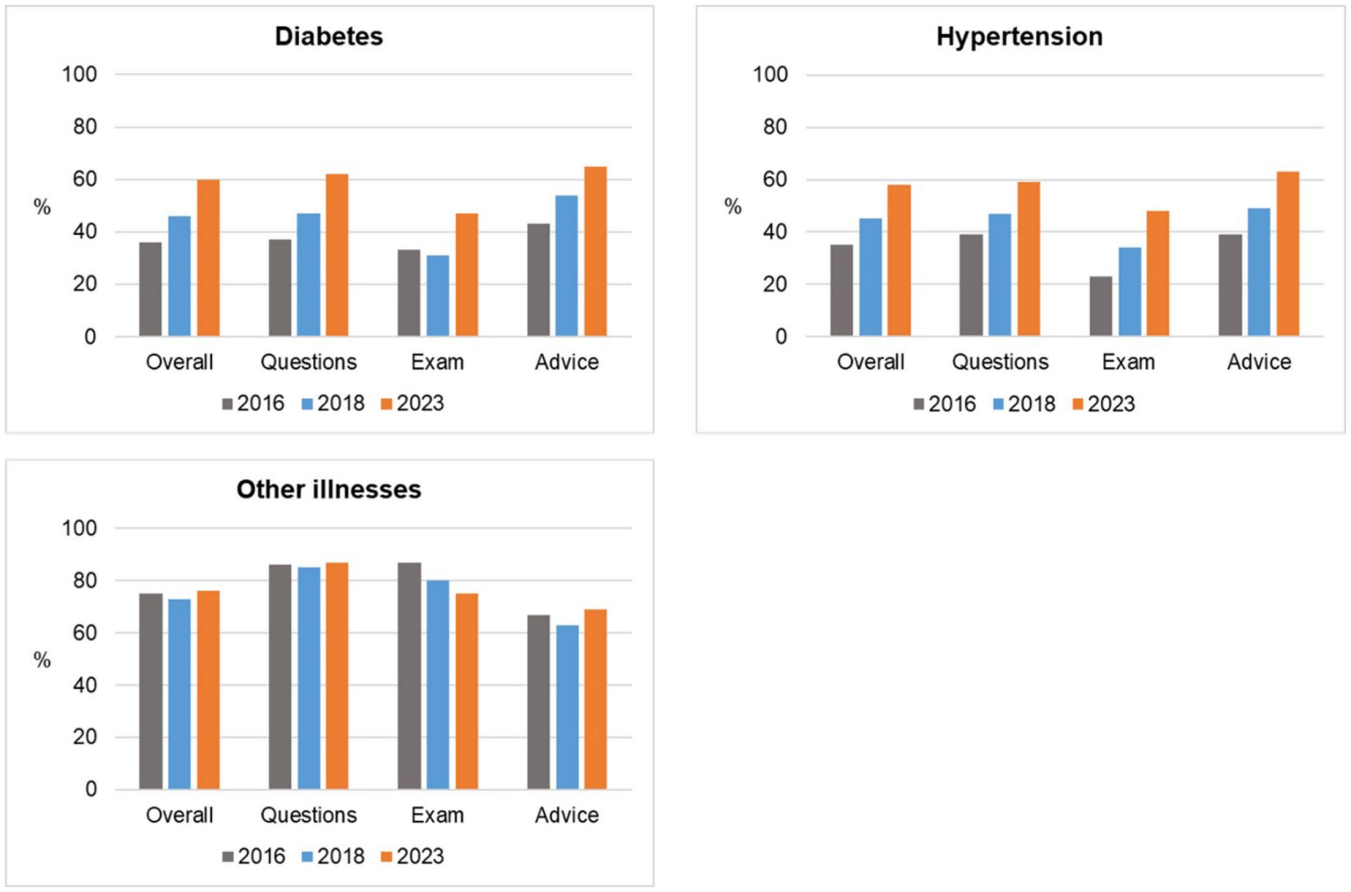
Disease-specific scores with sub-categories (2016, 2018, and 2023).

The disease-specific results of patients with diabetes mellitus, arterial hypertension and ‘other illnesses’ were further divided into the following sub-categories: (i) Questions—whether the doctor asked questions about the illness; (ii) Examination—whether the doctor conducted the examination appropriately; and (iii) Advice– whether the doctor advised and explained results, diagnosis and further steps sufficiently (Figure 4).

The clinical observations on diabetes and hypertension patients (*n* = 174 in 2016, *n* = 279 in 2018 and *n* = 81 in 2023) revealed a low adherence to procedural and treatment guidelines in 2016. Questions that the doctors were required to ask diabetic and hypertension patients during consultations covered topics such as whether the patient had any specific health complaints, had a sedentary lifestyle, problems with urine discharge, appetite, eye-sight, alcohol consumption and smoking. Examination steps that the doctors were required to perform during consultation of diabetic and hypertension patients included blood pressure checks, weight measurement and calculation of the body-mass index, examination of the eyes, the skin, mucus membranes, lymph nodes, ears, nose and thyroid glands. Advice that doctors are required to give to diabetic and hypertension patients during consultations included the results of any examinations, situation and diagnosis, prognosis, nutritional advice and the prevention and treatment of hypoglycaemia and other acute and chronic complications of diabetes, signs of extreme hypertension and importance of adherence to treatment, physical exercise and advice on contraceptives.

In 2018, save for a slight decrease in the percentage of examinations of diabetic patients (from 33% in 2016–31% in 2018), general improvements for the adherence to protocols were observed during consultations overall (from 36% in 2016–46% in 2018–60% in 2023). General improvements adhering to protocols were observed overall during consultations with patients with hypertension (from 35% in 2016–45% in 2018–58% in 2023). Overall, adherence to procedural and treatment guidelines for conditions other than hypertension and diabetes remained largely the same from 75% in 2016–73% in 2018–76% in 2023.

### Exit Interviews

**Figure 5:**
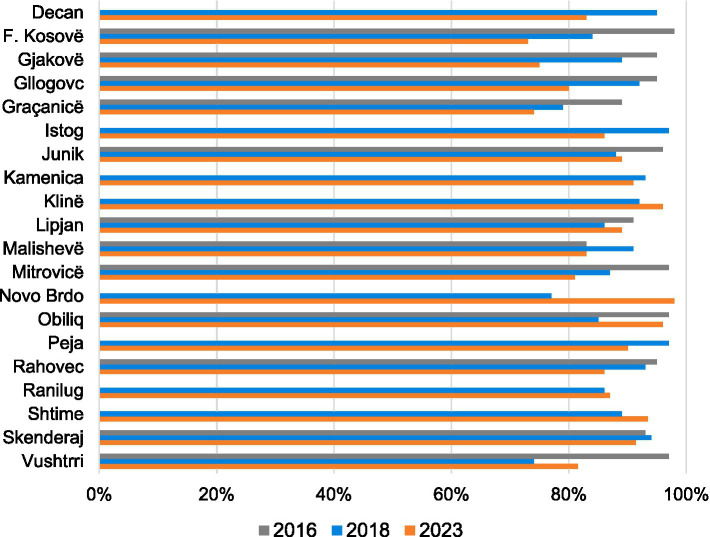
Exit interview scores (2016, 2018, and 2023).

In 2016, 716 exit interviews were conducted, 410 in MFMCs and 306 in FMCs, compared to 629 in 2018, 365 in MFMCs and 264 in FMCs. In 2023, 948 exit interviews were conducted, 574 in MFMCs and 432 in FMCs. The percentage of patients that reported not having visited a health centre in the 3 months prior to the survey increased from 21% in 2016 to 33% in 2018 and 33% in 2023. The overall experience of patients exiting facilities was positive; patients were mostly satisfied or very satisfied with the health services. In comparison to 2018, the ratio of very satisfied patients vs. satisfied patients increased in 2023 (Figure 5).

**Figure 6:**
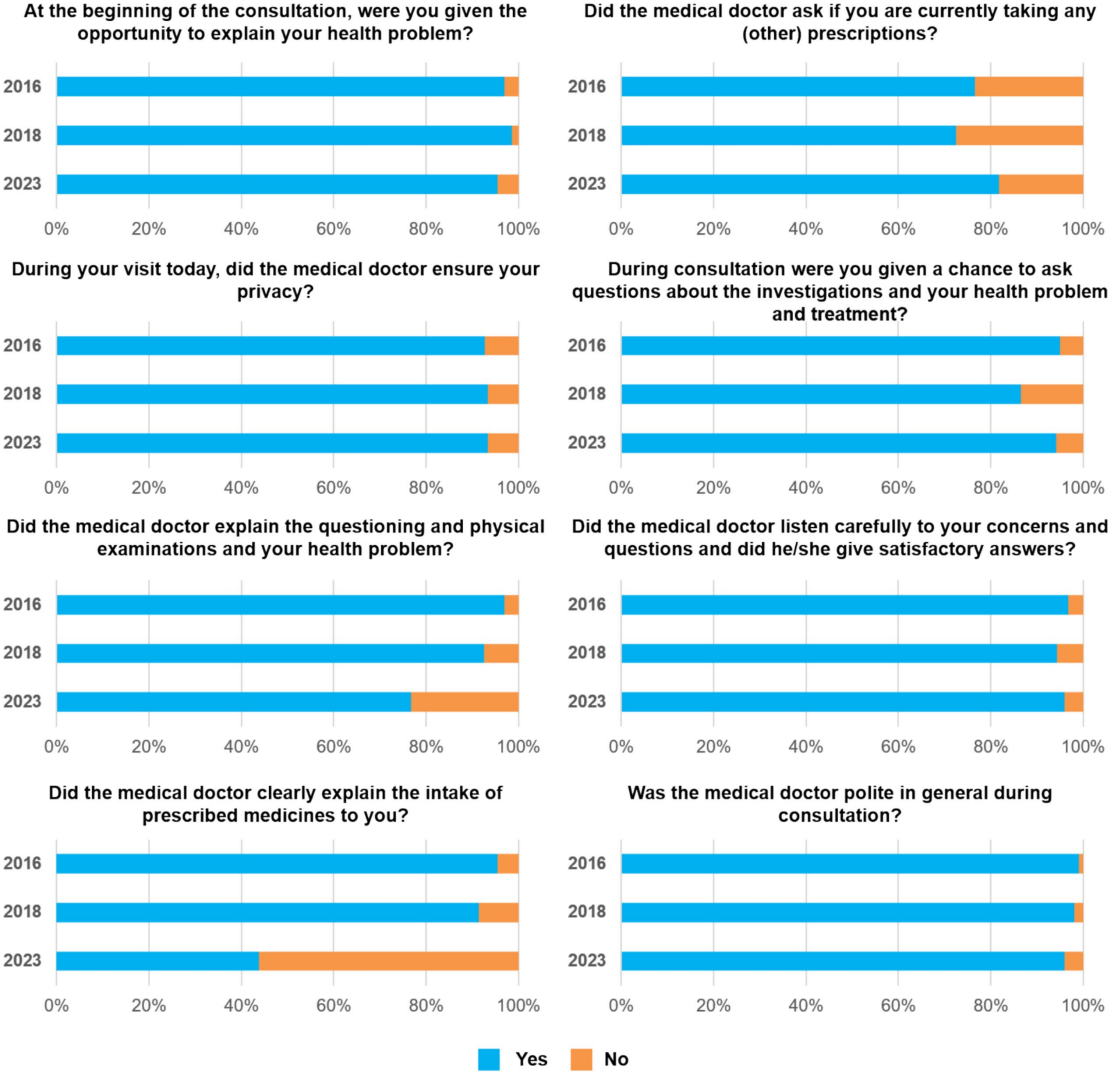
Exit interview scores of doctors’ behaviour indicators during consultation (2016, 2018, and 2023).

The overall patients service score markedly decreased in seven municipalities (Fushë Kosove, Gjakove, Gllogovc/Drenas, Malisheve, Rahovec, Decan and Istoq). A meaningful positive change, in terms of patient experience, could only be observed in Novo Brdo despite the declining infrastructure results from 2018 to 2023. During exit interviews, patients gave their perspectives of the doctors’ behaviour during consultation. These perspectives were measured over eight different indicators across all years, 2016, 2018 and 2023 (Figure 6). The majority of patients regarded the doctors’ behaviour positively (>60%) in all recorded years and in seven of the eight indicators. The noticeable negative exceptions were regarding the medication intake explanation, which became progressively worse from 96% in 2016 to 91% in 2018 and finally to 56% in 2023. The indicator regarding the doctors’ explaining the questioning, physical examination and health problems also headed in a downwards trajectory from 97% in 2016 to 93% in 2018 and 77% in 2023.

At the qualitative level, patients reported positive or negative changes on various aspects regarding consultations from different facilities between 2018 and 2023. The following are examples of key positive changes during the consultations: In Mitrovicë, Lipjan, Malishevë, Obiliq and Vushtrri the patient was given a chance to ask questions about the investigation, the health problem and treatment. In Mitrovicë, Malishevë and Vushtrri the doctor listened carefully to patients concerns and questions and gave a satisfactory answer and in Lipjan, Skenderaj, Vushtrri and Novo Brdo the doctor asked if the patient was currently taking prescription medication. Key negative aspects during consultations included; the doctor not explaining the questioning, physical examinations and the health problem in Gjakovë, Malishevë, Fushë Kosovë, Skenderaj, Skenderaj, Kamenica and Istog. In addition, the doctor did not explain the intake of prescribed medicine in all but seven municipalities.

An overview on the overall scores per municipality for each domain revealed that the overall infrastructure score increased between 2018 and 2023, in all municipalities except Novo Brdo (Table 2). Results from the clinical observations results vary, with many municipalities (*n* = 9) showing an increase in overall score trends and several others where the score decreased overall (*n* = 10). With few exceptions (Shtime, Ranilug, Novo Brdo and Klinë) the trend from the exit interviews was negative. In six municipalities, the decrease from 2018 to 2023 was statistically significant (Decan, Gjakovë, Gllogovc, Istog, Malishevë, Rahovec) while only Novo Brdo had a significant positive increase.

**Table 2:** Overall scores by municipality (2016, 2018, and 2023).

Municipalities	No of facilities	Infrastructure score [*n*^a^]			Clinical consultation score [*n* (95% CI)]			Exit interview score [*n* (95% CI)]		
		2016	2018	2023	2016	2018	2023	2016	2018	2023
Decan	2	n/a	49	67	n/a	41 (33–49)	63 (56–69)	n/a	95 (88–100)	82 (79–87)
Fushë Kosovë	3	61	64	75	74 (70–78)	70 (63–76)	69 (66–72)	98 (97–100)	84 (77–92)	73 (68–78)
Gjakovë	5	67	62	70	80 (77–84)	46 (42–50)	72 (69–75)	95 (92–98)	89 (86–93)	75 (69–80)
Gllogovc/Drenas	3	53	55	72	62 (62–63)	63 (60–66)	70 (67–74)	95 (93–96)	91 (88–94)	80 (77–83)
Gracanica^b^	3	70	65	71	58 (.-.)	55 (49–61)	n/a*	89 (.-.)	79 (72–85)	74 (67–80)
Istog	2	n/a	65	70	n/a	78 (74–82)	64 (52–77)	n/a	97 (95–99)	86 (77–95)
Junik	1	62	61	76	73 (.-.)	42 (27–57)	77 (74–80)	96 (.-.)	88 (80–96)	89 (.-.)
Kamenica	2	n/a	62	72	n/a	79 (75–83)	76 (72–80)	n/a	93 (87–98)	91 (86–96)
Klinë	1	n/a	56	75	n/a	79 (76–81)	65 (57–73)	n/a	92 (86–97)	96 (94–99)
Lipjan	5	44	49	67	59 (.-.)	70 (68–72)	82 (80–84)	91 (.-.)	86 (83–89)	89 (84–93)
Malishevë	3	44	53	58	54 (53–54)	63 (60–65)	34 (31–38)	83	91 (88–94)	83 (81–86)
Mitrovicë	6	54	64	68	75	81 (77–85)	86 (84–89)	97 (94–99)	87 (81–93)	81 (75–87)
Novo Brdo	2	n/a	60	55	n/a	75 (70–79)	64 (58–70)	n/a	77 (68–85)	97 (94–100)
Obiliq	2	59	63	66	71 (70–72)	83 (73–92)	76 (72–80)	97 (96–98)	85 (61–100)	96 (93–99)
Peja	3	n/a	62	66	n/a	72 (68–75)	75 (71–79)	n/a	94 (90–97)	90 (83–97)
Rahovec	3	58	62	71	70 (57–82)	52 (47–57)	62 (57–67)	95 (95–96)	93 (90–97)	86 (83–89)
Ranilug	1	n/a	60	62	n/a	75 (70–79)	69 (61–78)	n/a	86 (80–91)	87 (79–95)
Shtime	2	n/a	60	68	n/a	74 (67–81)	74 (70–78)	n/a	89 (84–94)	93 (89–97)
Skenderaj	3	52	57	66	62 (57–67)	80 (78–82)	72 (68–76)	93 (89–96)	94 (90–97)	91 (88–95)
Vushtrri	3	60	55	79	65 (63–67)	59 (53–66)	82 (78–85)	97 (95–98)	74 (64–85)	82 (77–86)
Overall	55	56	59	68	62 (61–63)	67 (66–68)	70 (68–71)	93 (91–94)	89 (88–90)	83 (82–85)

a 95% CIs not displayed due to small sample sizes.

b Upon request, in 2023, only facility infrastructure was assessed and exit interviews were performed.

**Figure 7:**
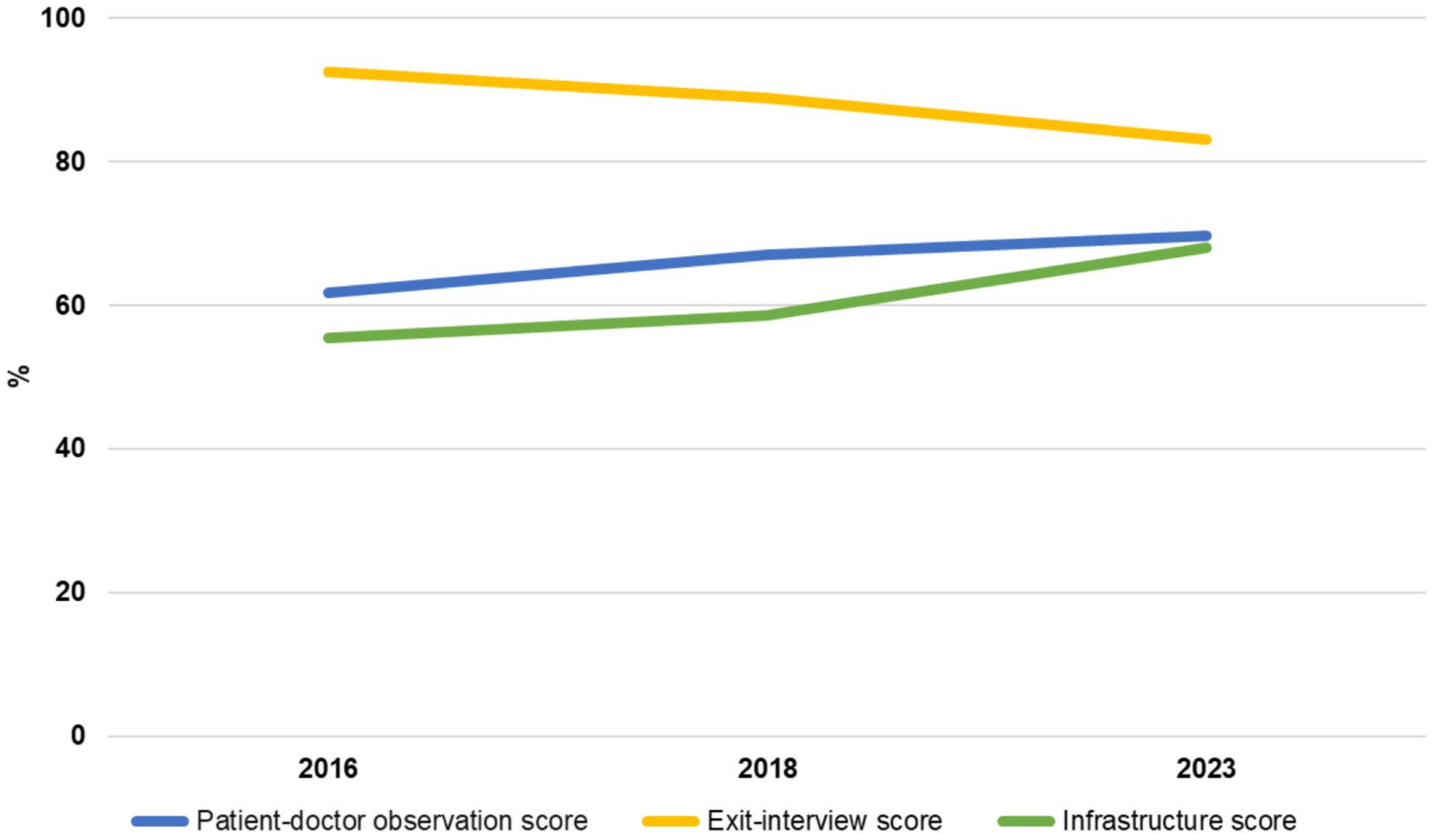
Mean overall scores by year (2016, 2018, and 2023).

A mean comparison was created to observe the overall infrastructure progress, progress of the doctor-patient performance and the exit interview scores for 2016, 2018 and 2023 (Figure 7). The infrastructure score showed slight improvement between 2016 and 2018 and then a marked improvement between 2018 and 2023. The quality of the doctor-patient interaction increased steadily over 2016, 2018 and 2023, whereas the patient satisfaction score decreased over all the years.

## Discussion

Results show disparities between municipalities in different domains. A combination of implementing interventions and systemic factors may have contributed to this.

Some cases faced specific issues, such as Malisheva (observing the lowest clinical observation score), which reported an abrupt loss of all its family physicians within 1 year. This may have disrupted service continuity and quality. In general, there are different ratios of physicians to patient service demands, which can impact service availability and quality in varying levels across the municipalities (31).

Municipalities also differ in their healthcare investments, which are influenced by varying health financing sources. Some generate higher municipal revenues, while others benefit from greater infrastructural investments depending on central-level financing and jointly established priorities. A detailed analysis of municipal health expenditures could reveal how differing investment priorities relate to the quality of healthcare services (32).

In addition, the timing of the intervention delivery may have contributed to differences in the quality of care. Some project supported interventions were implemented periodically rather than uniformly across municipalities. For those municipalities that received the full spectrum of support in 2022 only, more time may be needed to observe a stable effect across the domains. It is important to note that the COVID-19 pandemic also disrupted the timeline for some interventions particularly between 2020 and 2022.

The overall improvements in Kosovo’s healthcare infrastructure, from 56% in 2016 to 68% in 2023, are consistent with the global emphasis on the importance of healthcare infrastructure for delivering quality care (33, 34). Flaws in functional healthcare infrastructure, which include access issues and sub-standard medical care, can dissuade potential patients from seeking timely medical care jeopardising their own health and that of their community (35). Further, according to a review published in 2022, investments in physical infrastructure, among others, was considered an important factor toward attaining universal health coverage in many low- and middle-income countries (36, 37). Specifically, the overall hygiene indicator scores within the infrastructure survey were higher in 2016 than in 2018 but improved again in 2023. This positive trend in hygiene practices aligns with the global push for infection prevention and control (IPC), a priority in light of the COVID-19 pandemic. The improvement of the hygiene indicator in this study could be a consequence of the AQH project support in this area, a growing awareness of IPC measures due to a relevant AQH training module, which had been offered since 2017, and/or as an after effect of the rigorous hygiene requirements employed after the COVID-19 pandemic (38, 39).

A remaining matter of concern was the decreasing availability of medicines in about half of the municipalities between 2016 and 2023. In addition, both in MFMCs and FMCs, the availability of medicines was lower in 2023 than in 2016. The AQH project, however, did not have a mandate to support the availability of medicines, and this domain remained under the responsibility of the Ministry of Health (MoH).

The assessment of clinical practices and the dimension-specific sub-scores (questions, examination and advice), particularly in the management of NCDs like diabetes and hypertension, increased steadily over the intervention years, reaching about 60% for both diagnoses in 2023. This improvement reflects AQH’s support to the MoH in designing care pathways for NCDs and establishing clinical guidelines and protocols that are aligned to international best practices.

Several studies have shown that educational interventions, including training, are effective in promoting adherence to clinical practice guidelines (40). The increase in doctors’ knowledge and compliance with principles of clinical history and physical examinations could, therefore, be attributed to the AQH support that was given to the MoH for the training and roll-out of clinical practice guidelines for the treatment of diabetes and hypertension. One reason for inconsistent adherence to protocols among doctors may be the frequent turnover of healthcare providers in PHC facilities. Often, after graduation, doctors begin working in PHC centres before leaving to start their residency programs, leading to high staff turnover in PHC centres.

The observational scores for doctor’s hygiene, IPC measures increased steadily in 14 municipalities but remained low in many. The increase in doctors adhering to handwashing practices before and after procedures could be related to stringent COVID-19 hygiene measures impressed upon the health sector during the pandemic in addition to the continuous medical education on infection prevention provided by AQH project. Despite the mostly positive increases in IPC measures overall, it is clear that continuing professional development in this field is required in all municipalities to raise awareness to appropriate levels and to reduce healthcare-associated infections and infectious diseases, which should include both theoretical and practical learning activities (41).

Regarding the disease-specific sub-category diabetes, the overall score (including the anamnesis, examination and advice) increased by 24% from 2016 to 2023. The biggest increase over the same period was observed in the questions the doctors put to the patients sub-score (25%). The overall hypertension score increased by 23% from 2016 to 2023 with the largest increase in the examination sub-score (25%). The overall score for the sub-category other illnesses (other than diabetes or hypertension) increased by 1% from 2016 to 2023 and the examination sub-score decreased by 12%. However, despite only a slight increase in all categories except the examination sub-score, all sub-categories (questions, examination and advice) had a base value in 2016 above 60% and remained stable across all the years, 75, 73 and 76%, respectively. Comparing all sub-categories, diabetes and hypertension scores barely scratched the surface of 60% in all sub-categories even by 2023. The positive trends in adherence to guidelines related to diabetes and hypertension could be attributed to the support provided by the AQH project to their municipal partners. Adherence to clinical guidelines is essential for managing chronic conditions like diabetes and hypertension, which are leading causes of morbidity and mortality in Kosovo and globally (42). As observed in this study, the initial low adherence rates to diabetes protocols in 2016 (36%) are not unique to Kosovo but reflect broader trends especially in LMICs, where healthcare workers often struggle with limited training and high patient volumes (43–45). However, the significant improvements by 2023, especially the doubling of patients with hypertension that were examined, reflect the impact of targeted interventions and training programs aimed at improving clinical skills.

The overall positive experience of patients exiting the facilities could be accredited not just to the improvements in infrastructure and clinical practices but also the positive patient-provider relationship. Effective communication and empathy are key determinants of patient satisfaction, often more so than technical quality of care (46). However, the 48% drop in the clear explanation of medication intake highlights a critical gap in the patient-provider communication that needs to be addressed. This gap has been linked to the insufficient medical communication training seen in post-socialist health education systems (47). This could call for targeted training programs to improve these specific communication skills among doctors that appear to be lacking, which must be carefully adapted to local contexts and social structure (48).

The overall patients service score that significantly decreased in the seven municipalities, Fushë Kosove, Gjakove, Gllogovc/Drenas, Malisheve, Rahovec, Decan and Istog was interpreted as a positive trend in regards to the project objectives as this trend could indicate that patients had a more critical appreciation of health services after heavy interventions to increase patients’ awareness of their rights. Addressing the disparities in underperforming regions requires a multi-faceted approach that could include increased investments in human resources and targeted investments towards increasing the support and training of healthcare workers to ensure accountability in healthcare delivery.

### Limitations

Similar to the study limitations elsewhere (26, 27) medical students acted as observers for the clinical consultations and were trained and received clear instructions prior to data collection. However, the observers were not the same across the three surveys. In addition, while many observations can be controlled through training of the observers, some subjectivity on the level of the observation remains. For some questions, it was also up to the observers to judge on whether a denominator was applicable in a given situation. For example, mask wearing during a consultation on hypertension may have been perceived as “not applicable” and closing a consultation politely after issuing a prescription or a signature process was not necessarily perceived as real consultation. Equally, the frequency of a patient visiting may have influenced processes; medical doctors may see the same patient several times a week but take physical measures only once per week. Although efforts were made to introduce a greater degree of standardization, potential influences on the outcomes cannot be excluded.

The experimental design did not allow for linking the data from the clinical observations and the exit interviews. Separating the two processes, however, was necessary to ensure confidentiality and to avoid direct hesitancy and/or a biased response by the patients when having to provide a quality judgement if it can directly be linked to the personal doctor.

The study is limited to specific time measurement points. Many changes that took place during the COVID-19 peak period (2020–2022) may have affected the results, including disruptions to supply chains, staff training and other systemic adjustments.

Regarding the study instruments, although based on widely used model-based tools, they have not been formally validated in Kosovo. However, rigorous translation/back-translation, pilot testing for cultural clarity, reliance on prior Albanian and Kosovar experience, and alignment of clinical and infrastructure surveys with current Kosovar health legislation were ensured.

The study would benefit from patient clinical outcome data, which could be linked to the overall infrastructure and clinical improvements. However, due to lack of electronic health records in the national health system, it is not possible to access clinical data. Other confounders like comorbidities and education level were also not available, and since our analysis focused on descriptive statistics, we did not perform inferential modelling that would require controlling for confounding variables.

For health providers, the awareness of being observed by a third party, or knowing that exit interviews are taking place on the same day, suggests that a positive bias in their performance cannot be ruled out (Hawthorne effect) (49).

## Conclusion

The quality of care survey in PHC in Kosovo between 2016 and 2023 reveals marked progress in infrastructure, clinical practices, and hygiene standards, particularly in the management of NCDs. However, municipal disparities significantly impact overall progress and a consistency in achievements. Therefore, a national intervention is necessary to address the differences in municipal capacity and especially financial resources. Central requirements for standardized equipment and infrastructure must be continuously monitored and enforced, including through audits. In addition, the gap in doctor-patient communication and the uneven adherence to clinical protocols underscore the need for a continued and more focused approach to those areas. Advanced training of staff for protocol adherence is necessary, and quality monitoring of the protocols must be consistently reported at the municipal and central level. In addition, future studies can focus on the systemic administrative differences to better understand disparities at the local level. Kosovo’s healthcare reforms must prioritise equitable access to care, sustained investments in healthcare worker training, and improvements in patient-provider communication. Strengthening the healthcare system’s capacity to deliver high-quality, patient-centred care will be essential for Kosovo to achieve its broader health goals and align with EU healthcare standards.

## Data Availability

The datasets presented in this article are not readily available because of confidentiality considerations of the public health care institutions. Requests to access the datasets should be directed to shege.bahtiri@aqhproject.org.
